# Development and validation of the physical literacy scale for young and middle-aged patients with hypertension

**DOI:** 10.1186/s41043-025-01027-6

**Published:** 2025-09-30

**Authors:** Guiyue Ma, Xiaoqin Ma

**Affiliations:** 1 School of Nursing, Anhui University of Chinese Medicine, Hefei, Anhui China; 2 Key Laboratory of Geriatric Nursing and Health, Anhui University of Chinese Medicine, Hefei, Anhui China; 3 School of Nursing, Zhejiang Chinese Medical University, Hangzhou, Zhejiang China

**Keywords:** Hypertension, Physical literacy, Young and middle-aged, Validation, Measurement tools

## Abstract

**Background:**

In recent years, the significance of physical literacy in enhancing the well-being of patients with hypertension has gained increasing recognition. Physical literacy, defined as the motivation, confidence, physical competence, and knowledge and understanding necessary to engage in physical activities, plays a crucial role in promoting a healthy lifestyle. However, most existing studies have focused on the general population or specific subgroups, such as children, adolescents, and older adults, with limited attention to the unique needs of young and middle-aged patients with hypertension.

**Objectives:**

The study aimed to develop and validate the physical literacy scale for young and middle-aged patients with hypertension (PLS-YMPH). We investigated the reliability and validity of this scale to evaluate its quality, providing a valid tool for assessing physical literacy in this specific group.

**Methods:**

Initial items were developed through a literature review and face-to-face interviews. The item pool was modified based on the results of the two-round Delphi method. After forming the initial draft of the PLS-YMPH, items were screened using the critical ratio method, correlation coefficient method, Cronbach’s α coefficient method, and exploratory factor analysis. Finally, reliability and validity tests were conducted. The content validity, construct validity, discriminant validity, as well as content reliability, split-half reliability, and test-retest reliability of the scale were calculated.

**Results:**

The developed scale consists of 4 dimensions and 18 items. The Cronbach’s α coefficient for the overall scale was 0.943, with individual dimensions ranging from 0.917 to 0.946. The split-half reliability coefficient was 0.833, and the dimensions ranged from 0.919 to 0.947. The test-retest reliability coefficient was 0.854, with dimensions ranging from 0.805 to 0.959. The S-CVI/Ave for the scale was 0.918, and the values for each dimension were 0.925, 0.906, 0.896, and 0.948, all exceeding 0.80.

**Conclusions:**

The developed PLS-YMPH demonstrates good reliability and validity. It provides a valuable tool for assessing the physical literacy of this specific patient group, laying the groundwork for further research in this area.

**Supplementary Information:**

The online version contains supplementary material available at 10.1186/s41043-025-01027-6.

## Introduction

In recent years, the prevalence of hypertension has been rising alarmingly, particularly among younger populations [1]. Currently, hypertension affects approximately 1.28 billion people worldwide [2]. A particularly concerning trend is the decreasing age of hypertension onset. Younger individuals with hypertension face a greater burden of disease, including an increased susceptibility to cardiovascular problems, a higher risk of cognitive decline, and a greater likelihood of premature death [3]. This situation not only poses a significant threat to the health of young people but also places a growing strain on healthcare systems globally. Given their high long-term risk of cardiovascular disease, this demographic represents a key target for preventive strategies. To achieve the goals of “Healthy China 2030” and reduce the incidence of cardiovascular diseases, it is crucial to strengthen the management and control of hypertension in this group [4].

Key risk factors for patients with hypertension include physical inactivity, stress, obesity, high salt intake, and smoking or excessive alcohol consumption [5]. Promoting a healthy lifestyle, especially focusing on modifiable factors, is essential for managing hypertension in this population. Physical activity, a well-recognized non-pharmacological intervention, can significantly prevent cardiovascular diseases [6]. However, technological advancements have contributed to decreased physical activity and increased sedentary behavior [7]. The physical activity levels of Chinese patients with hypertension, particularly among the young and middle-aged population, are notably low [8]. Therefore, enhancing the physical activity levels of young and middle-aged patients with hypertension is a critical and urgent issue.

Physical literacy (PL) is a concept proposed by Margaret Whitehead in 2001, encompassing a comprehensive approach to engaging in physical activity throughout life. Rooted in existential and phenomenological philosophy, Whitehead’s framework defines PL as “the motivation, confidence, physical competence, knowledge, and understanding to value and take responsibility for engagement in physical activities for life” [9]. This theory expands on traditional fitness models by highlighting four interconnected domains [10]: (1) Cognition - Understanding the health benefits of movement and implementing self-regulatory strategies for managing hypertension. (2) Emotion - Fostering intrinsic motivation and building confidence to overcome barriers such as fatigue or time constraints. (3) Physical Competence - Developing motor skills that align with the capabilities of patients with hypertension. (4) Behavior - Converting knowledge and skills into sustainable activity patterns. The theory underscores the importance of valuing physical activity, health awareness, physical competence mastery, as well as fostering intrinsic motivation and self-confidence. Developing physical literacy is a dynamic process where motivation and confidence drive participation, while physical competence and knowledge provide a solid foundation. The key to enhancing physical literacy is consistent practice, which is reinforced through engaging physical activities. This improvement is especially crucial for patients with hypertension, as it encourages them to maintain regular physical activity [11].

Improving physical literacy can lead to increased activity levels, enhanced cardiopulmonary function, greater muscle strength, and better maintenance of a healthy weight and body fat ratio [12]. Additionally, it plays a crucial role in regulating blood pressure and lipid levels, helping to prevent the onset and progression of cardiovascular diseases [13]. Furthermore, high levels of physical literacy positively impacts mental health by reducing anxiety and depression, boosting self-confidence and enthusiasm, and enhancing overall work efficiency and quality of life. Therefore, it is essential to actively enhance physical literacy in this population.

Recent studies underscore the importance of physical literacy; however, existing scales primarily cater to children, adolescents, pregnant women, and the elderly. They often overlook the unique challenges and needs of young and middle-aged patients with hypertension [14]. Developing a physical literacy scale specifically for this specific group is crucial to addressing these gaps. This scale will assess the level of physical literacy among young and middle-aged patients, providing a scientific basis for targeted intervention strategies and encouraging greater participation in physical activities. Therefore, this study aims to achieve two objectives: (1) to create a valid instrument for measuring physical literacy tailored to young and middle-aged patients with hypertension, and (2) to evaluate the quality of this instrument by assessing its reliability and validity.

## Method

### Research design and participants

From December 2023 to January 2024, a convenience sampling approach was used to investigate patients at two community hospitals and five representative residential areas in Hefei City and Hangzhou City. These locations were selected to reflect diverse demographic characteristics and living environments. The sample size for the study was determined to be between 115 and 230 participants, which is 5 to 10 times the number of items in the initial scale [15]. The inclusion criteria for participants were as follows: (1) aged between 18 and 59 years [16]; (2) meeting the diagnostic criteria for hypertension as specified in the 2020 edition of the National Guidelines for the Prevention and Control of Hypertension at the Primary Level, which requires systolic blood pressure (SBP) of 140 mmHg or higher and/or diastolic blood pressure (DBP) of 90 mmHg or higher [17]; (3) having clear consciousness and unimpaired communication abilities. The exclusion criteria included: (1) individuals with severe cardiovascular and cerebrovascular diseases; (2) individuals with cognitive or communication impairments.

### Expert panel composition

A total of 16 experts were selected from four different disciplines to ensure methodological rigor. The group included four nursing experts specializing in hypertension care, each with peer-reviewed publications in hypertension management; four sports science experts skilled in assessing physical literacy and exercise physiology; four psychology experts experienced in scale development and psychometric testing; and four statistics experts specializing in scale validation. All experts held at least a master’s degree, and associate professor title, with ≥ 5 years of professional experience. Two rounds of the Delphi method were conducted to achieve consensus on item categorization, thus establishing content validity.

### Developing the first draft of the scale

The initial items of PLS-YMPH were developed through an integrated approach combining a literature review and qualitative interviews. A comprehensive search across databases like PubMed, Web of Science, SinoMed, and CNKI using keywords related to physical literacy yielded 2,443 articles, from which six Chinese and nine English questionnaires were selected for cross-cultural reference. Semi-structured interviews lasting 30 to 45 minutes with 15 patients with hypertension explored factors influencing physical activity, complemented by a focus group discussion involving seven team members to integrate literature-derived items. The iterative process resulted in an initial 24-item scale.

### Quality analysis of preliminary scale items

In a pre-survey of 200 participants with a 95% effective recovery rate, multiple psychometric methods were employed for item reduction. Specifically, through the critical ratio test using the 27th and 73rd percentile cut-offs, item-total correlation analysis with a deletion criterion of *r* < 0.3, evaluation of Cronbach’s α improvement upon item deletion, and exploratory factor analysis (EFA) showing a KMO measure of 0.92 and a Bartlett’s test *p* value < 0.001, six items were eliminated, resulting in an 18-item final scale (see Appendix 1).

### Assessment of face and content validity of the scale

The face validity of the PLS-YMPH was qualitatively evaluated through collaboration with ten patients. Feedback from patients led to the identification and correction of ambiguities related to item meanings, phrasing, scaling, grammatical errors, and item allocation. Question relevance was assessed using a 4-point Likert scale (1=not relevant to 4=highly relevant). The Content Validity Index (CVI) for each item was calculated as the proportion of specialists who rated it a 3 or 4. Following Polit and Beck’s criteria, a minimum acceptable CVI of 0.78 was established [18]. After the second round of the Delphi method, content validity indices were calculated. This included the Item-Content Validity Index (I-CVI) for each item, the Scale-Content Validity Index (S-CVI) for each dimension, and the average S-CVI (S-CVI/Ave) across all dimensions. The evaluation standards required an I-CVI of at least 0.78. After adjusting for random consistency among experts, the adjusted kappa value (K*) needed to exceed 0.74 to indicate good item content validity. An S-CVI/Ave of 0.90 or higher demonstrated satisfactory content validity for both individual dimensions and the overall scale [19].

### Assessment of the construct validity of the scale

The construct validity of the PLS-YMPH was assessed through EFA and confirmatory factor analysis (CFA). The required sample size for scale development was calculated to be between 5 and 10 times the number of items in the questionnaire [20]. Given that the sample size needed for CFA is typically more than 200 and considering potential issues such as response rates and invalid questionnaires during the study, the final sample size for the survey was increased by 20% based on the calculated size[21].

### Exploratory factor analysis

The various dimensions of the criteria were identified through an EFA using principal component analysis [22]. For this study, IBM SPSS Statistics version 26 was utilized for the EFA, applying Varimax rotation with Kaiser Normalization, given that the factor correlations were below 0.3 [23]. To evaluate the suitability of the sample for factor analysis, both the KMO measure and Bartlett’s test of sphericity were conducted. A KMO value exceeding 0.9 was considered satisfactory. Items with eigenvalues greater than one and factor loadings of 0.4 or higher were retained.

### Confirmatory factor analysis

In this study, construct validity was initially assessed through EFA and subsequently validated using CFA with Amos 28.0 (IBM Corp.) for data analysis [24]. The model fit indices used in this study included χ²/df, GFI, RMSEA, NFI, IFI, TLI, CFI, and RFI. A value of χ²/df less than 3 indicates a good fit, while an RMSEA value below 0.08 suggests an acceptable model fit. Specifically, RMSEA values under 0.05 indicate an excellent fit, while values between 0.05 and 0.08 indicate a good fit. Additionally, other fit indices are considered satisfactory if they exceed 0.9, with values above 0.85 deemed acceptable. Furthermore, CFA was used to evaluate the convergent validity of each dimension of the scale. Good convergent validity was established if standardized factor loadings exceed 0.45, composite reliability (CR) was greater than 0.7, and average variance extracted (AVE) was above 0.5.

### Discriminant validity and correlation analysis

Discriminant validity was assessed using the AVE. Good discriminant validity is established when the square root of the AVE for each dimension exceeds the correlation coefficients among the dimensions. This indicates that each dimension of the construct is distinct from the others and that the measurement instrument effectively differentiates between various concepts. Additionally, the correlation coefficients between the total scale score and the scores of each dimension were calculated to evaluate discriminant validity. Higher correlation coefficients between the dimensions and the total scale score reflect better consistency. The interpretation of correlation coefficients is as follows: a correlation coefficient of |r|=1 indicates a perfect correlation; |r| values between 0.70 and 1 signify a high correlation; values between 0.40 and 0.70 indicate a moderate correlation; values between 0.10 and 0.40 indicate a low correlation; and values below 0.10 indicate a negligible correlation.

### Assessment of reliability of the scale

#### Internal consistency reliability

Cronbach’s α coefficient is a measure of internal consistency reliability that indicates how well the items in a scale reflect the same construct. A higher value of Cronbach’s α indicates greater reliability of the scale. In this study, the internal consistency of both the overall scale and its dimensions was assessed by calculating Cronbach’s α coefficient.

#### Split-half reliability

Split-half reliability method involves dividing the items of the scale into two halves, typically by separating odd and even items. The correlation between the two halves was then calculated and adjusted using the Spearman-Brown formula. This method provides an estimate of the scale’s internal consistency by comparing the correlation between the two halves.

#### Test-retest reliability

Test-retest reliability measures the stability of a scale over time. To assess this, a randomly selected group of young and middle-aged patients with hypertension took the scale twice, with a two-week interval between administrations. By comparing the scores from these two tests, the reliability of the scale was assessed. Pearson correlation coefficients are calculated for scores from both administrations across each dimension as well as the overall scale score, reflecting the consistency of the scale over time.

### Scoring

The PLS-YMPH consists of four dimensions with a total of 18 items: Cognition (5 items), Emotion (3 items), Physical competence (5 items), and behavior (5 items). It use a 5-point Likert scale ranging from 1 (“Strongly Disagree”) to 5 (“Strongly Agree”). The total score ranges from 18 to 90, with higher scores indicating greater levels of physical literacy among patients.

## Results

### Baseline characteristics of participants

A total of 216 questionnaires were distributed, yielding 210 valid responses (97.2% response rate). The participants had a mean age of 48.27 ± 9.32 years, with 54.8% male and 52.4% aged 51–60 (Table 1).

**Table 1:** Basic information of patients with hypertension (*n* = 210)

Variables	Classification	Total (*n*)	Frequency (%)
Gender	Male	115	54.8
	Female	95	45.2
Age (years)	30～40	52	24.8
	41～50	48	22.9
	51～60	110	52.4
Marital status	Unmarried	51	24.3
	Married	159	75.7
Educational level	Primary school and below	18	8.6
	Junior middle school	41	19.5
	High school	40	19.0
	Bachelor degree or above	111	52.9
Occupation	Be unemployed	54	25.7
	Farming	4	1.9
	On the job	142	67.6
	Retire	10	4.8
Monthly per capita household income	<1000	14	6.7
	1000～3000	48	22.9
	3001～5000	52	24.8
	>5000	96	45.7
Family location	City	156	74.3
	Rural areas	54	25.7
Living situation	Live alone	16	92.4
	Live with family	194	7.6
Type of medical insurance	Self-payment	12	5.7
	New rural cooperative medical system	48	22.9
	Basic medical insurance for urban workers	102	48.6
	Basic medical insurance for urban residents	44	21.0
	Commercial medical insurance	4	1.9

### Formal scale validity test results

#### Content validity

The item-level CVI (I-CVI) ranged from 0.813 to 1.000, all exceeding the recommended threshold of 0.78. The scale-level S-CVI/Ave was 0.918, with the average S-CVI for each dimension being 0.925, 0.906, 0.896, and 0.948, all exceeding 0.80. Additionally, all items had K^*^ values greater than 0.74, indicating excellent content validity. Detailed results are shown in Table 2.

**Table 2:** Content validity of the scale

Item	Number of experts	Number of people with 4 or 3 points	I-CVI	Pc	K^*^
C1	16	16	1.000	0.0000	1.000
C3	16	15	0.938	0.0002	0.937
C4	16	14	0.875	0.0018	0.875
C5	16	15	0.938	0.0002	0.937
C6	16	14	0.875	0.0018	0.875
E1	16	15	0.938	0.0002	0.937
E4	16	14	0.875	0.0018	0.875
E5	16	13	0.813	0.0085	0.811
E7	16	14	0.875	0.0018	0.875
E8	16	15	0.938	0.0002	0.937
E9	16	16	1.000	0.0000	1.000
P3	16	15	0.938	0.0002	0.937
P5	16	14	0.875	0.0018	0.875
P6	16	15	0.938	0.0002	0.937
P7	16	14	0.875	0.0018	0.875
P8	16	14	0.875	0.0018	0.875
P9	16	14	0.875	0.0018	0.875
B1	16	15	0.938	0.0002	0.937
B3	16	15	0.938	0.0002	0.937
B5	16	16	1.000	0.0000	1.000
B6	16	15	0.938	0.0002	0.937
B7	16	14	0.875	0.0018	0.875
B8	16	16	1.000	0.0000	1.000

Note: I-CVI: Item-Content Validity Index; Pc: Probability value; K^*^: Adjusted kappa value

#### Construct validity

Building on content validity, construct validity was evaluated through EFA and CFA.

### Exploratory factor analysis

The KMO statistic for the study sample was 0.906, exceeding the threshold of 0.9, and Bartlett’s test of sphericity yielded a χ^2^ of 3700.633 (df = 153, *P* < 0.001), indicating statistical significance and fulfilling the criteria necessary for factor analysis. After extracting factors with eigenvalues greater than 1, the variances explained was 23.436%, 22.049%, 21.160%, and 14.517%, resulting in a cumulative variance of 81.162%, well above the 50% benchmark, which indicates that the factors account for a substantial portion of the total variance . All item factor loadings were greater than 0.4, as detailed in Tables 3 and 4.

**Table 3:** Explains the total variance

Composition	Initial eigenvalue			Extract the sum of squares of loads			Sum of squares of rotational loads		
	Total	Percentage of variance	Accumulation%	Total	Percentage of variance	Accumulation%	Total	Percentage of variance	Accumulation%
1	9.283	51.573	51.573	9.283	51.573	51.573	4.219	23.436	23.436
2	2.483	13.793	65.365	2.483	13.793	65.365	3.969	22.049	45.485
3	1.627	9.040	74.406	1.627	9.040	74.406	3.809	21.160	66.645
4	1.216	6.756	81.162	1.216	6.756	81.162	2.613	14.517	81.162

**Table 4:** Component matrix after rotation

Dimension	Item	Factor			
		1	2	3	4
Cognition	Moderate physical activity can promote blood circulation, control blood pressure levels, and reduce the risk of related complications.	0.841			
	Engaging in moderate-intensity aerobic exercise can lower blood pressure and improve heart function.	0.856			
	Excessive and intense physical activities, such as sprinting, are not conducive to maintaining stable blood pressure and increase the burden on the cardiovascular system.	0.857			
	It is advisable to avoid intense physical activities immediately after meals to minimize the potential impact on blood pressure.	0.857			
	Appropriate warm-up exercises before physical activity are helpful in preventing excessive fluctuations in blood pressure.	0.766			
Emotion	I value the benefits of moderate physical activity in managing blood pressure.				0.828
	I am interested in various physical activities for blood pressure control and am willing to try new exercise methods.				0.826
	If the weather is favorable, I am confident that I can maintain a certain amount of physical activity even when I feel lazy or am short on time.				0.801
Physical competence	I can engage in low- to moderate-intensity aerobic endurance exercises to effectively manage blood pressure.			0.681	
	I can perform stretching exercises for joints and muscles, including morning stretches, standing torso rotations, and lateral arm stretches, each lasting 10 to 30 seconds, to enhance flexibility and improve joint mobility.			0.749	
	I can perform various balance exercises, such as single-leg stands, balance walking, and heel-to-toe walking in a straight line, to reduce the risk of falling.			0.742	
	I can perform exercises for improving comprehensive functions like balance, agility, coordination, and gait, such as tai chi, yoga, table tennis, and badminton.			0.870	
	I can perform low- to moderate-intensity strength exercises, such as pushing, pulling, dragging, lifting, and pressing with 10 to 15 repetitions per set to improve muscle strength.			0.866	
Behavior	To maintain a balance between work and daily life, I prefer walking, cycling, or taking the stairs for transportation.		0.756		
	I engage in moderate-intensity aerobic exercises 3 to 5 times a week, averaging 30 minutes each time.		0.885		
	I perform resistance exercises at least twice a week, selecting 1 to 3 types of movements each time, completing 8 to 12 sets for each movement to further enhance muscle strength.		0.891		
	During breaks, I prefer engaging in moderate physical activity to replace prolonged sitting and promote blood circulation.		0.860		
	I regularly do housework, such as washing vegetables, cooking, mopping the floor, and doing laundry, to keep physically active.		0.793		

### Confirmatory factor analysis

#### Model fit

**Fig. 1:**
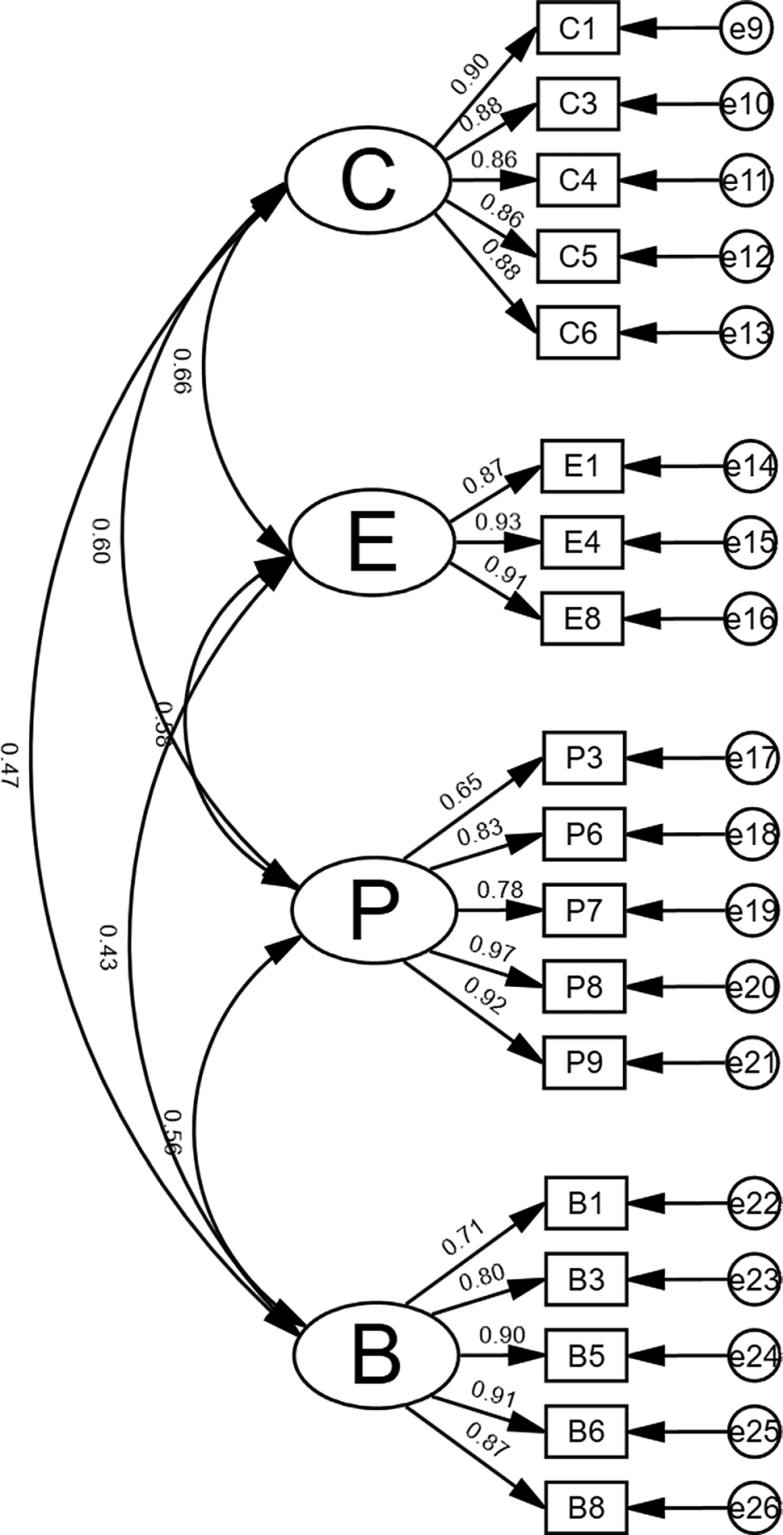
Confirmatory factor analysis model. Note: C: Cognition; E: Emotion; P: Physical competence; B: Behavior

A CFA was conducted using data from the formal survey. The results indicated that the χ²/df value was 2.060, which meets the criterion of being less than 3. The RMSEA was 0.071, falling below the threshold of 0.08. Additionally, the NFI, RFI, IFI, TLI, and CFI all reached values of 0.90 or higher. The GFI was 0.85, which is slightly below the optimal threshold of 0.90 but still considered acceptable in early-stage validation, especially when supported by other good fit indices [25]. No additional modifications were attempted to improve model fit, as the overall model fit met psychometric standards. Detailed results are shown in Table 5 and Fig. 1.

**Table 5:** Model fit

Fitting index	Measured value	Acceptable range
χ^2^	251.339	
df	122	
χ^2^/df	2.060	< 3
RMSEA	0.071	< 0.08
NFI	0.934	> 0.85
RFI	0.918	> 0.85
IFI	0.965	> 0.85
TLI	0.956	> 0.85
CFI	0.965	> 0.85
GFI	0.887	> 0.85

Note: χ²: Chi-square statistic; df: Degrees of freedom; χ²/df: Ratio of chi-square to degrees of freedom; RMSEA: Root Mean Square Error of Approximation; NFI: Normed Fit Index; RFI: Relative Fit Index; IFI: Incremental Fit Index; TLI: Tucker-Lewis Index; CFI: Comparative Fit Index; GFI: Goodness-of-Fit Index

#### Convergent validity

The convergent validity of each item dimension in the scale was assessed using CFA. The standardized factor loadings for each item were greater than 0.4, indicating statistical significance (*P* < 0.001). Additionally, the residuals were positive, which met the minimum standards. The AVE for each dimension was 0.699, 0.670, 0.616, and 0.703, all of which exceed the threshold of 0.5. The CR for each dimension was 0.921, 0.859, 0.888, and 0.922, all higher than the minimum requirement of 0.7, thus fulfilling the criteria for convergent validity. Detailed results are shown in Table 6.

**Table 6:** Convergent validity

Dimension	Item	Normalized factor load	S.E.	C.*R*.	*P*	AVE	CR
Cognition	C1	0.841				0.699	0.921
	C3	0.856	0.051	18.946	< 0.001		
	C4	0.857	0.056	17.837	< 0.001		
	C5	0.857	0.054	17.925	< 0.001		
	C6	0.766	0.048	19.087	< 0.001		
Emotion	E1	0.828				0.670	0.859
	E4	0.826	0.056	19.643	< 0.001		
	E8	0.801	0.054	18.767	< 0.001		
Physical competence	P3	0.681				0.616	0.888
	P6	0.749	0.120	10.605	< 0.001		
	P7	0.742	0.121	10.041	< 0.001		
	P8	0.870	0.131	11.885	< 0.001		
	P9	0.866	0.126	11.421	< 0.001		
Behavior	B1	0.756				0.703	0.922
	B3	0.885	0.098	12.416	< 0.001		
	B5	0.891	0.112	12.211	< 0.001		
	B6	0.860	0.103	12.507	< 0.001		
	B8	0.793	0.081	14.377	< 0.001		

Note: S.E.: Standard Error; C.R.: Critical Ratio; *P*: Probability value; AVE: Average Variance Extracted; CR: Composite Reliability

### Correlation analysis and discriminant validity

The correlation coefficients between the total scores of each dimension and the overall scale score were all greater than 0.7, with statistical significance (*P <* 0.01). This confirms a strong association and high internal consistency among the dimensions. To evaluate discriminant validity, a correlation analysis was conducted on the total scores of each dimension. The values were calculated using the formulas AVE = Σλ²/N and CR = (Σλ)²/((Σλ)² + Σ(1-λ²)), where λ represents the factor loading. The results showed that the absolute values of the correlation coefficients among the components ranged from 0.413 to 0.618, all of which were statistically significant, indicating consistent directional relationships. Moreover, the absolute values of the correlation coefficients between each component and the total scale ranged from 0.758 to 0.847, all of which were significant, further validating construct convergence. Discriminant validity was evaluated using the Fornell-Larcker criterion, which stipulates that the square root of each dimension’s AVE must exceed its correlations with all other dimensions. As shown in Table 7, the square roots of AVE for each dimension were as follows: cognition (0.836), emotion (0.819), physical competence (0.785), and behavior (0.838). This finding indicates that each dimension captures unique variance not explained by other constructs, thereby providing robust evidence for the discriminant validity of the PLS-YMPH.

**Table 7:** Correlation analysis and discriminant validity

	Cognition	Emotion	Physical competence	Behavior	Total
Cognition	0.836				
Emotion	0.618^**^	0.819			
Physical competence	0.563^**^	0.587^**^	0.785		
Behavior	0.413^**^	0.421^**^	0.513^**^	0.838	
Total	0.809^**^	0.774^**^	0.847^**^	0.758^**^	1.000

Note: ^**^: *P* < 0.01; The diagonal values are the square roots of the Average Variance Extracted (AVE)

### Reliability test results for the scale

After conducting validity assessments, we evaluated reliability as follows:

- Internal reliability: The overall Cronbach’s α coefficient for the scale was 0.943. The Cronbach’s α coefficient for each dimension range from 0.917 to 0.946, indicating strong internal consistency reliability both within the scale and across its dimensions.
- Split-Half Reliability: The split-half reliability for the total scale was 0.833. For each dimension, the split-half reliability ranged from 0.919 to 0.947, indicating that all values fall within an acceptable range.
- Test-Retest Reliability: The test-retest reliability coefficient for the total scale was 0.854. The coefficients for each dimension ranged from 0.805 to 0.959. These strong test-retest reliability results suggest that the scale demonstrates good stability. Detailed reliability results are presented in Table 8.

**Table 8:** Total amount table and reliability coefficient of each dimension(*n* = 210)

Dimension	Item	Internal reliability	Split-half reliability	Test-retest reliability
Cognition	5	0.946	0.947	0.959
Emotion	3	0.929	0.924	0.805
Physical competence	5	0.917	0.919	0.845
Behavior	5	0.930	0.926	0.867
Total	18	0.943	0.833	0.854

## Discussion

### Significance of developing the PLS-YMPH

The development of the PLS-YMPH is crucial considering the current state of physical literacy assessment and the specific needs of young and middle-aged patients with hypertension. Existing physical literacy scales, including the Physical Literacy Assessment Questionnaire (PPLA-Q) for 15- to 18-year-olds, the Adolescent Physical Literacy Questionnaire (APLQ) for 12- to 18-year-olds, and the Physical Literacy in Adults Scale (PLAS) for adults aged 18 to 75, are predominantly designed for healthy individuals [26–28]. Current scales do not adequately address the unique challenges faced by young and middle-aged patients with hypertension. The PLS-YMPH provides a critical solution by offering a specialized assessment tool designed for this group. It thoroughly evaluates various dimensions of patients’ physical literacy, including cognition, emotion, physical competence, and behavior related to physical activities. In the cognition dimension, the tool assesses patients’ understanding of how physical activity impacts the management of hypertension. Patients who are well-informed are more likely to adhere to effective exercise regimens, which can lead to improved blood pressure control [29]. The emotion dimension explores how a hypertension diagnosis influences patients’ attitudes toward physical activity. Feelings of anxiety or discouragement, which are common among these patients, can significantly affect their participation [30]. Recognizing these emotional barriers enables healthcare providers to offer more tailored support, potentially enhancing patients’ motivation and adherence to exercise [31].

Existing questionnaires have significant limitations [32, 33]. Some of these shortcomings stem from flaws in their development process, such as issues with content validity and stability. Furthermore, many questionnaires exhibit weaknesses in measurement properties, particularly regarding cross-cultural validity and measurement error [34]. These limitations prevent them from adequately considering the disease characteristics and physical literacy assessment needs of young and middle-aged patients with hypertension. This originality offers a more focused and relevant assessment compared to existing scales. Given the rising prevalence of hypertension among young and middle-aged individuals, the PLS-YMPH has significant implications. From a research perspective, the PLS-YMPH provides a valuable new tool for exploring the relationship between physical literacy and hypertension management in this specific patient group. By examining whether improving physical literacy leads to better blood pressure control and improved overall health outcomes, researchers can develop more effective interventions. Initiatives aimed at improving physical literacy may empower patients to manage their condition more effectively.

### Rigorous and standardized development process

The development of the PLS-YMPH followed a rigorous and systematic process, which is essential for ensuring its quality [35]. In the initial phase, a specialized team was formed, consisting of professionals from diverse backgrounds, including professors, nurses, and doctoral and master’s students with significant expertise in hypertension. By integrating perspectives from individuals with various professional experiences, the team ensured that the item development was methodologically sound and closely aligned with the theoretical framework of physical literacy. During the initial phase of constructing the item pool, a panel of 16 experts was invited to participate in the first round of the Delphi method. These experts, all holding master’s degrees or higher and specializing in hypertension and scale development, received draft versions of the scale items via email. This approach allowed them ample opportunity to thoroughly evaluate the relevance of the items and suggest any necessary modifications. Leveraging their extensive professional knowledge and practical experience, the experts evaluated the scale items from various perspectives. The research team then carried out a comprehensive analysis and synthesis of the consultation results. This iterative process served as a refinement mechanism, continuously improving and optimizing the scale items. Each cycle of feedback contributed to improving the scale, ensuring its accuracy in reflecting the physical literacy of patients.

### Excellent discrimination and homogeneity of scale items

When evaluating the quality of the PLS-YMPH, item analysis is a crucial step [36]. Its purpose is to comprehensively and deeply assess the quality of each item, ensuring the overall effectiveness and reliability of the scale. In this study, the critical ratio method and the Correlation coefficient method were employed to explore each item in depth. The critical ratio method assesses an item’s ability to distinguish between high- and low-scoring groups [37]. Significant differences in scores among these groups indicate effective differentiation. In this study, all items showed significant differences (*P* < 0.001), demonstrating strong discriminative power and confirming their ability to measure varying levels of physical literacy among patients accurately. The Correlation coefficient method evaluates item homogeneity by calculating the correlation between each item’s score and the total scale score [36]. High correlations suggest that the items consistently measure the same underlying construct, ensuring internal consistency. In this case, all items exhibited correlations ranging from 0.620 to 0.875 (*P <* 0.001), indicating strong coherence and alignment with the core concept of physical literacy. Overall, the PLS-YMPH shows excellent performance in items of discrimination and homogeneity. This reflects the scientific rigor of the scale’s development process and provides a solid foundation for its application in future research and clinical practice.

### Strong scale validity

The development and validation of the PLS-YMPH involved a systematic design and thorough verification process, which demonstrated its strong validity. Content validity, a crucial element of scale quality, was carefully established through the in-depth Delphi method and multiple rounds of questionnaire refinements [38]. This iterative process was crucial for aligning the content of the scale with the specific construct of physical literacy of young and middle-aged patients with hypertension. The I-CVI values were notably high, ranging from 0.813 to 1.000, surpassing the established benchmark of 0.78. Moreover, the overall S-CVI/Ave was 0.918, with each dimension’s average S-CVI exceeding 0.80. These results provide strong evidence of the scale’s content validity, indicating that the items in the PLS-YMPH are highly relevant to the concept of physical literacy for this patient group. This ensures that the scale effectively measures what it is intended to measure. Furthermore, construct validity, another important aspect of scale validity, was confirmed through factor analysis [39]. This statistical approach revealed a clear and meaningful factor structure among the scale items based on physical literacy theory. The satisfactory model fit, along with meeting the criteria for convergent validity and discriminant validity, further emphasized the scale’s strong construct validity. These findings suggest that the PLS-YMPH has a well-defined internal structure, allowing it to accurately represent the underlying construct of physical literacy. Consequently, the PLS-YMPH emerges as a reliable tool for precisely measuring physical literacy levels in this specific population, making a valuable contribution to the field of hypertension research and patient care.

### Robust scale reliability

In this study, a thorough reliability analysis was conducted on a scale designed to evaluate the physical literacy of young and middle-aged patients with hypertension. Internal consistency reliability was assessed using Cronbach’s α coefficient, a widely accepted metric for evaluating the relationships among items on the scale. The Cronbach’s α coefficient of 0.943 indicated a high level of internal consistency. This suggests that the items on the scale are strongly correlated, reflecting excellent overall reliability. Notably, this value exceeds those found in previous studies [32, 40], emphasizing the strength of the scale’s internal structure. The split-half reliability coefficient for the total scale was 0.833. This value suggests a high level of consistency between the two halves of the scale, which further supports its internal consistency. By dividing the scale items and examining the correlation between the two subsets, it is possible to assess whether the scale consistently measures the construct across different groupings of items. Test-retest reliability was evaluated using the Intraclass Correlation Coefficient (ICC). This involved administering the scale to the same group of participants twice, with a two-week interval between the two administrations. The test-retest reliability coefficient of 0.854 indicates that the PLS-YMPH demonstrates good short-term stability over a two-week interval. This suggests that physical literacy scores tend to remain relatively consistent when no interventions or significant lifestyle changes occur. However, it is important to note that long-term fluctuations are still possible. Hypertension management often requires dynamic adjustments in physical activity behaviors influenced by factors such as disease progression, seasonal changes, or personalized interventions. Consequently, future longitudinal studies that track physical literacy scores over extended periods (e.g., 6 to 12 months) are recommended to assess both its temporal stability and its responsiveness to clinical or behavioral interventions. Such studies could help clarify whether physical literacy is a stable trait or a modifiable state within this population [41]. In conclusion, the scale exhibits strong reliability. Its high internal consistency and strong test-retest reliability provide robust support for its credibility and effectiveness.

### Implications for practice

The PLS-YMPH provides valuable guidance for healthcare workers in both hospital and community settings. This scale is an essential tool for assessing the physical literacy of young and middle-aged patients with hypertension. By using the PLS-YMPH, healthcare workers can effectively identify patients with lower physical literacy scores, which may result from various factors, such as a lack of knowledge about the benefits of physical activity for managing hypertension, negative emotions toward exercise, or limited physical abilities. Once these patients are identified, healthcare workers can create and implement targeted physical literacy interventions. For those who score low in the knowledge-related aspects of the scale, educational materials about the positive impact of physical activity on blood pressure control can be provided. If patients show signs of low motivation or confidence, healthcare workers can offer encouragement and support. Based on the assessment results related to physical competence, healthcare workers can recommend appropriate physical activities. For patients with lower physical abilities, gentle exercises such as walking or simple stretching routines can be suggested, with intensity gradually increased as physical condition improves. By using the PLS-YMPH to guide these interventions, healthcare workers can effectively promote increased physical activity among patients. This, in turn, can lead to improved blood pressure management and an overall improvement in patients’ health and quality of life.

### Limitations

This study has several limitations. Firstly, the convenience sampling method employed in Hefei and Hangzhou may limit the generalizability of the findings. Although efforts were made to recruit participants from various demographic backgrounds, the sample may not fully represent young and middle-aged patients with hypertension across different regions or socioeconomic groups. Future studies should adopt more rigorous sampling techniques, such as stratified or random sampling, to enhance sample representativeness and improve external validity. Secondly, while the CFA demonstrated acceptable model fit overall, the GFI was slightly below the ideal threshold [42]. Although other fit indices supported the model’s validity, this indicates room for improvement in future validation efforts. Enhancements could be made by refining item wording or by using larger sample sizes (≥ 300) in future validation rounds [43]. Thirdly, the cross-sectional design precludes causal inference regarding the dynamic relationship between physical literacy and hypertension management. Longitudinal studies tracking participants over 6 to 12 months are necessary to evaluate the scale’s responsiveness to interventions. Lastly, the scale’s applicability in non-Chinese cultural contexts has not yet been tested, highlighting the need for cross-cultural validation in future research. To address these limitations, subsequent research should focus on the following: (1) implementing stratified sampling across multiple provinces to enhance sample representativeness, (2) refining the scale’s structural validity, particularly improving the GFI, (3) adopting longitudinal designs to assess predictive validity, and (4) conducting cross-cultural validation in various cultural settings. These efforts would help establish the PLS-YMPH as a reliable and generalizable tool for assessing physical literacy in young and middle-aged patients with hypertension.

## Conclusion

This study focuses on the systematic development and validation of the physical literacy scale for young and middle-aged patients with hypertension. The key conclusions are as follows: Firstly, the use of the Delphi method and iterative item refinement ensured that the scale possesses robust content validity. This was achieved by considering the multi-level structure of physical literacy and the specific needs of this patient demographic. Secondly, through statistical analysis, the internal structure of the scale was verified, revealing a clear and theoretically consistent factor structure that aligns with the framework of physical literacy. This provides a solid theoretical foundation for practical application. Lastly, in comparison to measures used in other relevant areas, this scale offers a more comprehensive and integrated assessment of physical literacy. It reflects a holistic perspective on the physical literacy of young and middle-aged patients with hypertension. In summary, the development of this scale is both scientific and systematic, demonstrating strong content and construct validity. It serves as a reliable tool for assessing physical literacy in young and middle-aged patients with hypertension, and it holds significant potential for clinical and research applications.

## Supplementary Information

Below is the link to the electronic supplementary material.

Supplementary Material 1

## Data Availability

The datasets generated and/or analyzed during the current study are not publicly available due to the sensitive nature of the questions asked in this study. Additionally, the survey respondents were assured raw data would remain confidential and would not be shared. The data is available from the corresponding author on reasonable request.

## References

[1] Sabapathy K, Mwita FC, Dauya E, et al. Prevalence of hypertension and high-normal blood pressure among young adults in zimbabwe: findings from a large, cross-sectional population-based survey[J]. Lancet Child Adolesc Health. 2024;8(2):101–11. 10.1016/S2352-4642(23)00287-0. 38070533 10.1016/S2352-4642(23)00287-0 PMC7617873

[2] Wandile PM. Hypertension and comorbidities: A silent threat to global health[J]. Hypertens Comorbidities. 2024;1(1):1–7. 10.46439/Hypertension.1.001.

[3] He R, Wei F, Hu Z, et al. Self-management in young and middle-aged patients with hypertension: a systematic review and meta-synthesis of qualitative studies[J]. Syst Reviews. 2024;13(1):254. 10.1186/s13643-024-02665-3. 10.1186/s13643-024-02665-3 PMC11453001 39369232

[4] Wang R, Wang Y, Lu J, et al. Forecasting cardiovascular disease risk and burden in China from 2020 to 2030: a simulation study based on a nationwide cohort[J]. Heart. 2025;111(5):205–11. 10.1136/heartjnl-2024-324650. 39638429 10.1136/heartjnl-2024-324650 PMC11874356

[5] Wójcik M, Alvarez-Pitti J, Kozioł-Kozakowska A, et al. Psychosocial and environmental risk factors of obesity and hypertension in children and adolescents—a literature overview[J]. Front Cardiovasc Med. 2023;10:1268364. 10.3389/fcvm.2023.1268364. 38054100 10.3389/fcvm.2023.1268364 PMC10694215

[6] Pastore MC, Cavigli L, Olivoni G, et al. Physical exercise in hypertensive heart disease: from the differential diagnosis to the complementary role of exercise[J]. Int J Cardiol. 2024;132232. 10.1016/j.ijcard.2024.132232. 10.1016/j.ijcard.2024.132232 38844090

[7] Sengkey SB, Sengkey MM, Tiwa TM, et al. Sedentary society: the impact of the digital era on physical activity levels[J]. J Public Health. 2024;46(1):e185–6. 10.1093/pubmed/fdad163. 10.1093/pubmed/fdad163 37622252

[8] Wang J-G, Zhang W, Li Y, et al. Hypertension in china: epidemiology and treatment initiatives[J]. Nat Reviews Cardiol. 2023;20(8):531–45. 10.1038/s41569-022-00829-z. 10.1038/s41569-022-00829-z 36631532

[9] Whitehead M. The concept of physical literacy[J]. Eur J Phys Educ. 2001;6(2):127–38. 10.1080/1740898010060205.

[10] Barratt J, Dudley D, Stylianou M, et al. A conceptual model of an effective early childhood physical literacy pedagogue[J]. J Early Child Res. 2024;22(3):381–94. 10.1177/1476718X231219580.

[11] Grauduszus M, Wessely S, Klaudius M, et al. Definitions and assessments of physical literacy among children and youth: a scoping review[J]. BMC Public Health. 2023;23(1):1746. 10.1186/s12889-023-16680-x. 37679785 10.1186/s12889-023-16680-x PMC10486121

[12] Nezondet C, Gandrieau J, Nguyen P, et al. Perceived physical literacy is associated with cardiorespiratory fitness, body composition and physical activity levels in secondary school students[J]. Children. 2023;10(4):712. 10.3390/children10040712. 37189960 10.3390/children10040712 PMC10136585

[13] Jiang T, Zhao G, Fu J, et al. Relationship between physical literacy and cardiorespiratory fitness in children and adolescents: a systematic review and meta-analysis[J]. Sports Med. 2024;1–13. 10.1007/s40279-024-02129-7. 10.1007/s40279-024-02129-7 PMC11947022 39579330

[14] Holler P, Carl J, Van Poppel MN, et al. Development of the perceived physical literacy questionnaire (PPLQ) for the adult population[J]. J Exerc Sci Fit. 2023;21(4):424–33. 10.1016/j.jesf.2023.09.003. 38028984 10.1016/j.jesf.2023.09.003 PMC10661355

[15] Sun Z, Xu Y. Medical Statistics[M]. BeiJing: People’s Medical Publishing House; 2002.

[16] Zhao J, Zeng L, Liang G, et al. Higher systemic immune-inflammation index is associated with sarcopenia in individuals aged 18–59 years: a population-based study[J]. Sci Rep. 2023;13(1):22156. 10.1038/s41598-023-49658-1. 38092854 10.1038/s41598-023-49658-1 PMC10719257

[17] Pallarés-Carratalá V, Ruiz-García A, Serrano-Cumplido A, et al. Prevalence rates of arterial hypertension according to the threshold criteria of 140/90 or 130/80 mmhg and associated cardiometabolic and renal factors: SIMETAP-HTN study[J]. Medicina. 2023;59(10):1846. 10.3390/medicina59101846. 37893564 10.3390/medicina59101846 PMC10608132

[18] Polit D, Beck C. Essentials of nursing research: appraising evidence for nursing practice[M]. Lippincott Williams & Wilkins; 2020.

[19] Ma G, Zhong Z, Duan Y, et al. Development and validation of a self-quantification scale for patients with hypertension[J]. Front Public Health. 2022;10:849859. 10.3389/fpubh.2022.849859. 35646756 10.3389/fpubh.2022.849859 PMC9132107

[20] Zi W, Song J, Kong W, et al. Tirofiban for stroke without large or medium-sized vessel occlusion[J]. N Engl J Med. 2023;388(22):2025–36. 10.1007/s43678-025-00915-4. 37256974 10.1056/NEJMoa2214299

[21] Pacewicz CE, Hill CR, Chun H, et al. Confirmatory factor analysis in kinesiology journals with explicit measurement focus[J]. Meas Phys Educ Exerc Sci. 2024;28(2):146–71. 10.1080/1091367X.2023.2270466.

[22] Howard MC, Henderson J. A review of exploratory factor analysis in tourism and hospitality research: identifying current practices and avenues for improvement[J]. J Bus Res. 2023;154:113328. 10.1016/j.jbusres.2022.113328.

[23] George D, Mallery P. IBM SPSS statistics 26 step by step: A simple guide and reference[M]. Routledge; 2019.

[24] Collier J. Applied structural equation modeling using AMOS: basic to advanced techniques[M]. Routledge; 2020.

[25] Sathyanarayana S, Mohanasundaram T. Fit indices in structural equation modeling and confirmatory factor analysis: reporting guidelines[J]. Asian J Econ Bus Acc. 2024;24(7):561–77. 10.9734/ajeba/2024/v24i71430.

[26] Mota J, Martins J, Onofre M. Portuguese physical literacy assessment questionnaire (PPLA-Q) for adolescents (15–18 years) from grades 10–12: development, content validation and pilot testing[J]. BMC Public Health. 2021;21:1–22. 10.1186/s12889-021-12230-5. 34844566 10.1186/s12889-021-12230-5 PMC8628133

[27] Mohammadzadeh M, Sheikh M, Houminiyan Sharif Abadi D et al. Design and psychometrics evaluation of adolescent physical literacy questionnaire (APLQ)[J]. Sport sciences for health, 2022, 18(2): 397–405. 10.1007/s11332-021-00818-8 10.1007/s11332-021-00818-8 PMC8379033 34457071

[28] Naylor A, Flood A, Barnett LM, et al. Development of the physical literacy in adults scale (PLAS)[J]. J Sports Sci. 2024;42(12):1099–111. 10.1080/02640414.2024.2383486. 39046323 10.1080/02640414.2024.2383486

[29] Dobrowolski P, Prejbisz A, Szyndler A, et al. Physician–patient partnership—can it help increase adherence to the therapeutic recommendations in cardiovascular disease?[J]. Arterial Hypertens. 2024;28:50–70. 10.5603/ah.103488.

[30] Balakrishnanpillai J, Kesavadev J, Saboo B. Harmony in health: A narrative review exploring the interplay of mind, body, and diabetes with a special emphasis on emotional Stress[J]. J Diabetol. 2024;15(2):123–30. 10.4103/jod.jod_132_23.

[31] Fernandes JB, Fernandes S, Domingos J, et al. Motivational strategies used by health care professionals in stroke survivors in rehabilitation: a scoping review of experimental studies[J]. Front Med. 2024;11:1384414. 10.3389/fmed.2024.1384414. 10.3389/fmed.2024.1384414 PMC11133544 38813377

[32] Alipour-Anbarani M, Ghaffari M, Montazeri A, et al. Development and psychometric of a physical literacy questionnaire for young adolescents (16–18 years of age): A mixed-method study[J]. Shiraz E-Medical J. 2023;24(9):e138738. 10.5812/semj-138738.

[33] Barnett LM, Jerebine A, Keegan R, et al. Validity, reliability, and feasibility of physical literacy assessments designed for school children: a systematic review[J]. Sports Med. 2023;53(10):1905–29. 10.1007/s40279-023-01867-4. 37341907 10.1007/s40279-023-01867-4 PMC10504218

[34] Sum RK, Cheng C-F, Wallhead T, et al. Perceived physical literacy instrument for adolescents: A further validation of PPLI[J]. J Exerc Sci Fit. 2018;16(1):26–31. 10.1016/j.jesf.2018.03.002. 30662489 10.1016/j.jesf.2018.03.002 PMC6323161

[35] Devellis RF, Thorpe CT. Scale development: theory and applications[M]. Sage; 2021.

[36] Izah SC, Sylva L, Hait M. Cronbach’s alpha: A cornerstone in ensuring reliability and validity in environmental health assessment[J]. ES Energy Environ. 2023;23:1057. 10.30919/esee1057.

[37] Galanis P, Katsiroumpa A, Vraka I, et al. The quiet quitting scale: development and initial validation[J]. AIMS Public Health. 2023;10(4):828–48. 10.3934/publichealth.2023055. 38187899 10.3934/publichealth.2023055 PMC10764970

[38] Rusticus S. Content validity[M]. Springer; 2024.

[39] Tavakol M, Wetzel A. Factor analysis: a means for theory and instrument development in support of construct validity[J]. Int J Med Educ. 2020;11:245–7. 10.5116/ijme.5f96.0f4a. 33170146 10.5116/ijme.5f96.0f4a PMC7883798

[40] Diao Y, Wang L, Chen S, et al. The validity of the physical literacy in children questionnaire in children aged 4 to 12[J]. BMC Public Health. 2024;24(1):869. 10.1186/s12889-024-18343-x. 38515090 10.1186/s12889-024-18343-x PMC10956319

[41] Vrinten J, Van Royen K, Pabian S, et al. Development and validation of a short nutrition literacy scale for young adults[J]. Front Nutr. 2023;10:1008971. 10.3389/fnut.2023.1008971. 37020809 10.3389/fnut.2023.1008971 PMC10067712

[42] Finch WH. Using fit statistic differences to determine the optimal number of factors to retain in an exploratory factor analysis[J]. Educ Psychol Meas. 2020;80(2):217–41. 10.1177/0013164419865769. 32158020 10.1177/0013164419865769 PMC7047263

[43] Da Rocha NS, Power MJ, Bushnell DM, et al. The EUROHIS-QOL 8-item index: comparative psychometric properties to its parent WHOQOL-BREF[J]. Value Health. 2012;15(3):449–57. 10.1016/j.jval.2011.11.035. 22583455 10.1016/j.jval.2011.11.035

