# Supplementary material for: Development and validation of the physical literacy scale for young and middle-aged patients with hypertension
Source: J Health Popul Nutr. 2025 Sep 30;44:338. doi: 10.1186/s41043-025-01027-6 (PMC12486499; doi:10.1186/s41043-025-01027-6)
Supplement: Supplementary file 1 — Supplementary Material 1 [file 41043_2025_1027_MOESM1_ESM.docx]

**Appendix 1**

**Basic information of experts**

A total of 16 experts from four fields (nursing, sports science, psychology, and statistics) were invited. The sample included 13 females (81.3%) and 3 males (18.8%), with a mean age of 43.19±6.52 years. Seven experts (43.8%) held a doctoral degree, and the rest had a master's degree. Most experts (37.5%) had 11-15 years of work experience. In terms of professional titles, 6 experts (37.5%) were Professors, and the others were associate professors. The details are shown in Table S1.

Table S1 Basic characteristics of experts (n=16)

| **Expert** | **Gender** | **Age (years)** | **Education** | **Work Experience (years)** | **Professional Title** |
| --- | --- | --- | --- | --- | --- |
| 1 | Female | 55 | Master | >20 | Professor |
| 2 | Female | 37 | Doctor | 11-15 | Associate Professor |
| 3 | Female | 53 | Doctor | >20 | Professor |
| 4 | Female | 46 | Doctor | 16-20 | Professor |
| 5 | Male | 36 | Master | 6-10 | Associate Professor |
| 6 | Female | 35 | Master | 6-10 | Associate Professor |
| 7 | Female | 38 | Master | 11-15 | Associate Professor |
| 8 | Female | 41 | Master | 11-15 | Associate Professor |
| 9 | Male | 39 | Master | 11-15 | Associate Professor |
| 10 | Female | 47 | Master | >20 | Associate Professor |
| 11 | Female | 52 | Doctor | >20 | Professor |
| 12 | Female | 42 | Doctor | 11-15 | Associate Professor |
| 13 | Male | 37 | Master | 6-10 | Associate Professor |
| 14 | Female | 40 | Doctor | 11-15 | Associate Professor |
| 15 | Female | 43 | Doctor | 16-20 | Professor |
| 16 | Female | 50 | Master | >20 | Professor |

**Literature search strategy**

Databases: SinoMed, Web of Science, PubMed, Embase, Cochrane Library, CINAHL, CNKI, Wanfang, VIP

Keywords (English/Chinese):
"physical literacy", "survey", "questionnaire", "assessment", "scale", "validity", "reliability", etc.

Screening Process:

Total articles: 2443

→ Duplicates removed: 1664

→ Title/abstract screening: excluded 1587

→ Full-text review: included 55

→ Questionnaires selected: 22 (6 Chinese, 9 English)

**Semi-structured interview**

Sample Questions:

“Do you think there is a relationship between physical activity and hypertension control? If so, what kind of relationship is it?”

“What measures do you think can encourage people with hypertension like you to participate more actively in physical activities?”

“When you do physical exercise, do you choose exercises that can promote joint and muscle stretching, as well as enhance balance and comprehensive functions?”

“After being diagnosed with hypertension, have you ever tried to improve your physical condition through some physical exercises?”

“What do you think are the obstacles in your daily life that prevent you from engaging in physical activities?”

**Focus group composition**

One professor (nursing),

Two clinical nurses (hypertension specialty),

One doctoral student (hypertension management),

Three master’s students (cardiovascular chronic disease management).

**Quality analysis of preliminary scale items**

Tables S2 Item reduction details

| Method | Criteria | Items removed |
| --- | --- | --- |
| Critical ratio test | *p* > 0.05 (*t*-test) | - |
| Item-total correlation | r < 0.3 | B7 |
| Cronbach’s α coefficient | Cronbach’s α coefficient increases when items are removed | B7 |
| Exploratory factor analysis | Factor loading < 0.5 or cross-loading | E5, E7, E9, P5 |
